# rs2841277 (*PLD4*) is associated with susceptibility and rs4672495 is associated with disease activity in rheumatoid arthritis

**DOI:** 10.18632/oncotarget.19419

**Published:** 2017-07-18

**Authors:** Wei-Chiao Chen, Wen-Chang Wang, Yukinori Okada, Wei-Pin Chang, Yii-Her Chou, Hui-Hua Chang, Jin-Ding Huang, Der-Yuan Chen, Wei-Chiao Chang

**Affiliations:** ^1^ Institute of Clinical Pharmacy and Pharmaceutical Sciences, College of Medicine, National Cheng Kung University, Tainan, Taiwan; ^2^ The Ph.D. Program for Translational Medicine, College of Medical Science and Technology, Taipei Medical University, Taipei, Taiwan; ^3^ Master Program for Clinical Pharmacogenomics and Pharmacoproteomics, School of Pharmacy, Taipei Medical University, Taipei, Taiwan; ^4^ Department of Clinical Pharmacy, School of Pharmacy, Taipei Medical University, Taipei, Taiwan; ^5^ Department of Statistical Genetics, Graduate School of Medicine, Osaka University, Osaka, Japan; ^6^ School of Health Care Administration, College of Management, Taipei Medical University, Taipei, Taiwan; ^7^ Department of Urology, Kaohsiung Medical University Hospital, Kaohsiung, Taiwan; ^8^ Department of Urology, School of Medicine, College of Medicine, Kaohsiung Medical University, Kaohsiung, Taiwan; ^9^ School of Pharmacy, College of Medicine, National Cheng Kung University, Tainan, Taiwan; ^10^ Department of Internal Medicine and Medical Education, Taichung Veterans General Hospital, Taichung, Taiwan; ^11^ Faculty of Medicine, National Yang Ming University, Taipei, Taiwan; ^12^ Ph.D. Program in Translational Medicine, National Chung Hsing University, Taichung, Taiwan; ^13^ Institute of Biomedical Science and Rong Hsing Research Center for Translational Medicine, National Chung Hsing University, Taichung, Taiwan; ^14^ Center for Biomarkers and Biotech Drugs, Kaohsiung Medical University, Kaohsiung, Taiwan; ^15^ Department of Pharmacy, Taipei Medical University-Wanfang Hospital, Taipei, Taiwan

**Keywords:** rheumatoid arthritis (RA), phospholipase D family, member 4 (PLD4), rs2841277, rs4672495

## Abstract

Rheumatoid arthritis (RA) is one of the most common autoimmune diseases, can lead to long-term joint damage, chronic pain, and loss of motor function in the hands, and may share some common genetic factors with other autoimmune disorders, such as ankylosing spondylitis (AS). Many single-nucleotide polymorphisms (SNPs) were reported by genome-wide association studies (GWASs) of RA, but some of them have not been examined in the Taiwanese population. In this study, for 15 SNPs reported in previous RA and AS GWASs, we investigated their association with RA in a Taiwanese population. Based on 334 RA patients recruited from the Taichung Veterans General Hospital and 16,036 healthy subjects from the Taiwan Biobank (TWB) project, we observed that subjects having minor allele C at rs2841277 (*phospholipase D family, member 4* (*PLD4*)) have lower susceptibility of RA, compare to those having genotype TT (Odds ratio (OR) = 0.6, *p* = 3.0 × 10^−6^). Among the RA patients, we observed that subjects having GG at rs4672495 have a lower proportion of severe RA, compare to other subjects (OR = 0.09, *p* = 5.6 × 10^−3^). Results of a bioinformatics approach showed that rs2841277 is able to influence expression of *LINC00638* and *AHNAK2* and rs4672495 is able to influence the expression of *B3GNT2*. In summary, this study replicated an association of rs2841277 with RA susceptibility and showed an AS-associated SNP, rs4672495, is associated with RA activity in the Taiwanese population.

## INTRODUCTION

Rheumatoid arthritis (RA) is one of the most common autoimmune diseases, with a prevalence of 0.3%∼1.1% in population with European ancestry and 0.1%∼0.5% in those with Asian ancestry [1]. It is also a chronic inflammatory disease that can lead to long-term joint damage, chronic pain, and loss of motor function in the hands [2]. The disease is female predominant, with a 3:1 ∼ 4:1 female: male ratio [3]. Sera from the majority of RA patients contain autoantibodies, such as rheumatoid factor (RF) [4] or anti-citrullinated protein/peptide antibodies (ACPAs) [5], and ACPAs are now recognized as more highly specific for RA than RF [5, 6].

The cause of RA is still unclear. Both genetic and environmental factors are involved in its development and the estimated heritability of RA is approximately 60% [7]. Several genetic regions were reported to be associated with RA. The major histocompatibility complex (MHC) is a well-known region [8]. The human leukocyte antigen (HLA), especially HLA-DRB1, is the strongest susceptibility locus to RA beyond ethnicity [9, 10]. Besides HLA-DRB1, the disease susceptibility could be influenced by other loci within MHC, including amino acid position 9 in HLA-B, amino acid position 9 in HLA-DPB1, and amino acid position 77 in HLA-A [11, 12]. But MHC regions only explain around 12% of the total heritability of RA [13]. Genes involved in regulating peripheral tolerance were found have effects on the development of RA, such as *PTPN22* C1858T (rs2476601) [14], *CTLA-4* A49G [15], and *PDCD-1* (rs36084323) [16], and *PD-L1* 6777G was associated with the prevalence of rheumatoid nodules [17].

Recently, many non-MHC regions were found to be associated with RA by genome-wide association studies (GWASs) or meta-analyses of GWASs in various populations [18–21]. Some RA-associated loci reported by previous GWASs have been replicated in the Taiwan population. For example, *CCR6* was identified as an RA-associated gene in both Japanese and European populations [18, 19], and the same susceptibility locus has been successfully replicated by our previous study in a Taiwanese RA population [22]. Recently, through a meta-analysis of GWASs, Okada et al. (2012) indicated that nine new loci were associated with RA in a Japanese population [21], which have not been confirmed in the Taiwanese population. One purpose of this study is to investigate whether the polymorphisms reported by Okada et al. (2012) [21] are associated with RA in a Taiwan population.

Accumulating evidences indicated that RA may share some susceptibility genes with other autoimmune disorders [21], including ankylosing spondylitis (AS) [23, 24]. RA and AS are autoimmune articular diseases with different phenotypes and AS is mainly affecting the lumbar spine and sacroiliac joints and may exhibit peripheral arthritis [25]. More than 25 loci have been identified to be associated with AS [26]. A study by Sirota et al. compared genetic variation profiles of six autoimmune disease and found RA and AS fall into the same class [23]. Our previous studies showed that a polymorphism of *ORAI1* (rs7135617) is associated with both AS and RA in a Taiwanese population [27, 28]. Recently, for seven single-nucleotide polymorphisms (SNPs) reported by AS GWASs in several populations, we examined their association with AS in a Taiwanese population [29]. In addition to the RA-associated polymorphisms reported by Okada et al. (2012) [21], we will investigate whether the seven SNPs examined by our previous AS study [29] are associated with RA in the Taiwan population.

In this study, based on a group of RA patients collected from the Taichung Veterans General Hospital and a group of controls from the Taiwan biobank (TWB) project [30], we applied a two-stage approach to investigate association of the SNPs from Okada et al. (2012) [21] and Wen et al. (2014) [29] (Table 1) with susceptibility of RA in a Taiwanese population. Firstly, we identified SNPs whose genotype/allele distributions are different between RA patients and controls; then, we examined association of the identified SNPs with RA susceptibility under different genetic models. Based on the RA patients, the same approach was used to study association of these SNPs with disease activity and autoantibodies including ACPAs and RF.

**Table 1:** The single-nucleotide polymorphisms (SNPs) analyzed in this study

Class	SNP	Position (hg38) (bp)	Candidate gene	Allele		Minor allele frequencies (MAF)			HWE
				Major	Minor	EUR	JPT	TWB	
1	rs11900673	chr2:62225526	*B3GNT2*	C	T	0.13	0.33	0.18	0.28
1	rs657075	chr5:132094425	*CSF2*	G	A	0.13	0.38	0.25	0.46
1	rs12529514	chr6:14096427	*CD83*	T	C	0.06	0.13	0.23	0.78
1	rs2233434	chr6:44265183	*NFKBIE*	T	C	0.04	0.22	0.16	0.52
1	rs10821944	chr10:62025330	*ARID5B*	T	G	0.31	0.33	0.27	0.59
1	rs3781913	chr11:72662452	*PDE2A-ARAP1*	A	C	0.56	0.29	0.28	0.74
1	rs2841277	chr14:104924668	*PLD4*	T	C	0.52	0.30	0.42	0.24
1	rs2847297	chr18:12797695	*PTPN2*	A	G	0.32	0.35	0.30	0.29
2	rs11209032	chr1:67274409	*IL23R*	G	A	0.33	0.41	0.49	0.86
2	rs4672495	chr2:62294109		T	G	0.32	0.12	0.17	0.86
2	rs10865331	chr2:62324337		G	A	0.38	0.27	0.49	0.60
2	rs27434	chr5:96793809	*ERAP1*	A	G	0.79	0.52	0.47	0.40
2	rs3734523	chr6:25925759	*SLC17A2*	G	A	0.09	0.00	0.03	0.38
2	rs13202464	chr6:31376806	*HLA-B*	A	G	0.06	0.18	0.07	0.55
2	rs13210693	chr6:109277761		G	A	0.53	0.56	0.48	0.97

EUR, European; JPT, Japanese in Tokyo; TWB, Taiwan biobank; HWE, *p* value for Hardy-Weinberg equilibrium test in TWB.

SNPs of class 1 and class 2 were extracted from Okada et al. (2012) [21] and Wen et al. (2014) [29], respectively.

MAFs of EUR and JPT were extracted from NCBI; MAFs of TWB were obtained from the Taiwan View website (https://taiwanview.twbiobank.org.tw/index). Miner allele was determined by MAF of TWB.

## RESULTS

### Sample characteristics

In total, 334 RA patients were recruited for this study (Table 2). Among these patients, the median age is 59.0 (with IQR [48–66]) years; 278 (83.2%) are female, and 263 (78.7%) are RF (+). Two hundred and sixty-six patients (79.6%) have available ACPA status and 193 of them are positive. Measure of the 28-joint disease activity score (DAS28) is available for 290 patients and the median is 6.26 (with IQR [5.62-6.91]). According to DAS28, 290 subjects have available status of disease activity and 263 of them are severe.

**Table 2:** Basal characteristics of the 334 patients with rheumatoid arthritis (RA)

Characteristics	n (%)/Median [IQR]
Age (years)	59.0 [48–66]
Gender	
Female	278 (83.2)
Male	56 (16.8)
RF	
Positive (+)	263 (78.7)
Negative (−)	70 (21.0)
Unknown	1 (0.3)
ACPA	
Positive (+)	193 (57.8)
Negative (−)	73 (21.8)
Unknown	68 (20.4)
DAS28 (N = 290)	6.26 [5.62-6.91]
Disease activity	
Severe	263 (78.7)
Moderate	27 (8.1)
Unknown	44 (13.2)

IQR, interquartile range; RF, rheumatoid factor; ACPA, anti-cyclic citrullinated peptides antibody; DAS28, disease activity score by 28 joints. Severe, DAS28 score was larger than 5.1; Moderate, DAS28 score was between 3.2 and 5.1.

Up to the end of March 2017, TWB project has released genotype information of 16,036 healthy subjects through the Taiwan View website (https://taiwanview.twbiobank.org.tw/index) [30]. These 16,036 subjects were used as controls in this study. For each of the 15 SNPs analyzed, the obtained genotype counts of these controls were in Hardy-Weinberg equilibrium (HWE) (Table 1).

### SNP rs2841277 is associated with RA susceptibility

To investigate the association of polymorphisms with RA susceptibility, first, we compared the genotype and allele distributions between groups of RA patients and controls for each SNP and results were summarized in Supplementary Table 1. Among the 15 SNPs examined, rs2841277 (*PLD4*) revealed significant difference in both genotypic distribution (*p* = 8.3 × 10^−6^; *q* = 0.0012) and allelic distribution (*p* = 2.9 × 10^−6^; *q* = 0.0008). For other SNPs, no significant difference was observed. Then we investigated the association of rs2841277 (*PLD4*) with RA susceptibility under different genetic models (Table 3). The most significant association was observed under dominant model (*p* = 3.0 × 10^−6^) and the estimated OR is 0.6. This result indicates that subjects having minor allele C at rs2841277 (*PLD4*) have lower susceptibility of RA, compare to those having homozygous genotype TT. Similar results for rs2841277 (*PLD4*) were found in ACPA-positive patients and RF-positive patients. Specifically, compared to subjects having genotype TT, the OR of subjects carrying allele C among ACPA- and RF-positive patients is 0.56 (*p* = 6.6 × 10^−5^) and 0.57 (*p* = 6.1 × 10^−6^), respectively.

**Table 3:** Association of rs2841277 with rheumatoid arthritis susceptibility

Model	Genotype	Frequency (%)		OR (95% CI)	*P* value
		Case	Control		
Genotypic	CC	12.0	17.8	0.50 (0.35-0.70)	6.5E-05
	TC	41.7	48.2	0.64 (0.50-0.80)	1.1E-04
	TT	46.3	34.0	Reference	—
Dominant	CC+TC	53.7	66.0	0.60 (0.48-0.74)	3.0E-06
	TT	46.3	34.0	Reference	—
Recessive	CC	12.0	17.8	0.63 (0.45-0.88)	6.2E-03
	TC+TT	88.0	82.2	Reference	—
Trend test					3.3E-06

### SNP rs4672495 is associated with RA activity

In addition to susceptibility, we further assessed association of polymorphisms with activity of RA. In our study, 290 RA patients have available DAS28 score. According to EULAR criteria, 263 and 27 of these 290 patients were classified into groups of severe and moderate, respectively, and no patients were classified into groups of mild and remission. Therefore, for association with disease activity, the comparisons were conducted between groups of severe and moderate RA activity. For each SNP, the comparisons of genotype and allele distributions between severe and moderate RA patients were summarized in Supplementary Table 2. Among these comparisons, two SNPs, rs11209032 and rs4672495, revealed difference in genotype distribution (*p* = 0.040 and 0.0031, respectively). Since the corresponding *q*-values are 0.54 and 0.084, respectively, only the letter passes the threshold of significance when considering multiple testing (q<0.1) and the association of rs4672495 with disease activity was examined under different genetic models (Table 4). The most significant association was observed under recessive model (*p* = 5.6 × 10^−3^) and the significance remained even after applying the Bonferroni correction. The estimated OR is 0.09, which indicates a lower proportion of severe RA patients among subjects having GG at rs4672495, compare to other subjects.

**Table 4:** Association of rs4672495 with rheumatoid arthritis activity

Model	Genotype	Frequency (%)		OR (95% CI)^†^	*P* value^†^
		Severe	Moderate		
Genotypic	GG	1.1	11.1	0.12 (0.02-0.65)	1.4E-02
	TG	30.8	11.1	3.25 (0.94-11.25)	6.3E-02
	TT	68.1	77.8	Reference	—
Dominant	TG+GG	31.9	22.2	1.70 (0.66-4.39)	2.7E-01
	TT	68.1	77.8	Reference	—
Recessive	GG	1.1	11.1	0.09 (0.02-0.49)	5.6E-03
	TT+TG	98.9	88.9	Reference	—
Additive				1.02 (0.47-2.22)	9.6E-01

^†^Adjusted for gender and age.

### Association of polymorphisms with autoantibodies

RF and ACPAs are important autoantibodies in RA. Therefore, we also examined the association of polymorphisms with RF and ACPA. As shown in Supplementary Table 3, two SNPs, rs11900673 (*B3GNT2*) and rs2841277 (*PLD4*), revealed difference in allele distributions between ACPA (+) and ACPA (−) patients (*p* = 0.026 and 0.035, respectively). However, both tests have *q* = 0.5, which does not pass the threshold of significance for multiple testing. Furthermore, as shown in Supplementary Table 4, no SNP revealed difference in genotype/allele distributions between RF (+) and RF (−) patients.

### Studies of tissue expression quantitative trait loci (eQTLs) of rs2841277 and rs4672495

From data of the GTEx portal (Table 5), we found that rs4672495 could influence the expression of the *B3GNT2* (beta-1,3-N-acetylglucosaminyltransferase 2) gene in whole blood (*p* = 2.0 × 10^−26^). We also found that rs2841277 could influence expressions of the *C14orf79* (chromosome 14 open reading frame 79), *LINC00638* (long intergenic non-protein coding RNA 638), and *AHNAK2* (AHNAK nucleoprotein 2) genes.

**Table 5:** Expression Quantitative trail loci (eQTL) results from Genotype-tissue expression (GTEx)

SNP ID	Gencode ID(ENSG00000-)	Gene symbol	*p* value	Effect size	Tissue	Actions
rs2841277	140104.9	*C14orf79*	1.10E-08	0.29	Cells-Transformed fibroblasts	CC<CT<TT
	140104.9	*C14orf79*	2.10E-08	0.44	Brain-cortex	CC<CT<TT
	140104.9	*C14orf79*	6.60E-07	0.25	Skin-sun exposed (lower leg)	CC<CT<TT
	140104.9	*C14orf79*	2.30E-06	0.47	Brain-cerebellum	CC<CT<TT
	140104.9	*C14orf79*	8.30E-06	0.29	Esophagus-muscularis	CC<CT<TT
	258701.1	*LINC00638*	2.20E-05	−0.30	Artery-aorta	CC>CT>TT
	258701.1	*LINC00638*	2.80E-05	−0.25	Artery-tibial	CC>CT>TT
	258701.1	*LINC00638*	5.40E-05	−0.20	Thyroid	CC>CT>TT
	185567.6	*AHNAK2*	3.00E-05	−0.22	Esophagus-muscularis	CC>CT>TT
rs4672495	170340.10	*B3GNT2*	2.00E-26	0.34	Whole Blood	GG<GT<TT

## DISCUSSION

Because the major symptom of RA is chronic synovitis of multiple joints, which leads to bone erosion and joint destruction, a worsening of quality of life, and restrictions of activities of daily living, clarifying the characteristics of RA is important. Elucidation of pathogenic mechanisms will provide the potential therapeutic targets. Using GWASs or a candidate gene approach to detect susceptibility genes would also help uncover the pathophysiology underlying RA. In addition, many studies focusing on the disease severity of RA were conducted.

A large proportion of the identified risk loci for autoimmune diseases are related to innate immunity, or immune cell (T-cells and B-cells) activation pathways, differentiation, regulating peripheral tolerance, or involved cytokine signaling. The major histocompatibility complex (MHC) is a well-known region associated with RA [8], but it only explain around 12% of the total heritability in susceptibility to RA [13]. On the other hand, many non-MHC regions have been found to be associated with RA by recent GWASs [18–21]. Furthermore, a portion of RA loci have been shown to be specific to ethnicity. For example, the association of rs2075876 (*AIRE*) with RA reported in Japanese population [31] was replicated in Han Chinese population [32], but not in Caucasian population [33]. Therefore, any reported RA-associated polymorphisms deserve to be replicated in different populations. Recently, we have replicated the association of rs3093024 (*CCR6*) with RA in the Taiwanese population [22]. In this study, we successfully replicated an association of rs2841277 with RA in a Taiwan population, which was originally reported in the Japanese population [21].

More than 100 markers and loci have been associated with RA [34]. However, thus far, the detected markers cannot fully clarify the genetics of RA, which indicates that many susceptibility genes have yet to be identified. Accumulating evidences indicated that RA may share some susceptibility genes with other autoimmune disorders [23, 24]. Several studies have associated some RA-associated polymorphisms with other autoimmune diseases, such as systemic lupus erythematosus (SLE), AS, and Graves’ disease [21, 35]. Therefore, it is a reasonable approach to identify new RA-associated polymorphisms among the polymorphisms that have been associated with other autoimmune diseases.

RF and ACPA are two major autoantibodies present in sera from the majority of RA patients. But RF is recognized as being nonspecific for RA, as it may be found in other autoimmune diseases and immunological conditions [36]. Nevertheless, ACPA is a highly specific autoantibody for RA, which recognizes a broad range of citrullinated peptides and approximately 80% of RA patients with exhibited this autoantibody [5, 6]. Autoimmunity to citrullinated proteins is an important factor in the RA pathogenesis [37]. Although the specificity of RF has little diagnostic significance for RA, a high titer of RF suggests a poor RA prognosis [38, 39]. Therefore, we also examined association of SNPs with ACPA and RF in this study.

In this study, for the first time, we showed that rs2841277 in *PLD4* was significantly associated with RA susceptibility and potentially associated with autoantibodies of ACPA in a Taiwanese population. We observed that allele C of rs2841277 is associated with lower susceptibility to RA and this observation is consistent with the result of Okada et al. (2012) in Japanese RA populations, which reported T of rs2841277 is a risk allele for RA [21]. *PLD4* is a transmembrane glycoprotein, and it is a member of the phospholipase family without phospholipase D activity, as reported in 2010 [40]. A study of the white matter of mice found that *PLD4* was expressed in early postnatal microglia and the spleen [40], but phenotypes of *Pld4*-deficient mice have not been reported. Moreover, little is known about the distribution or expression of *PLD4* in human. Although the functions of *PLD4* are also poorly understood, one study indicated that it is involved in the phagocytosis of microglia [41]. In addition, Yoshikawa and coworkers reported that PLD4 expressed around the marginal zone in the spleen might support the function of PLD4 in immunologic systems [40]. According to results of previous studies, *PLD4* loci were reported to be associated with RA, and also associated with systemic lupus erythematosus (SLE) and systemic sclerosis (SSc) [21, 42]. These evidences suggest a role of *PLD4* in autoimmune diseases.

Another finding of this study is association between an AS-associated SNP, rs4672495, and activity of RA. The rs4672495 polymorphism is contained in an intergenic region on chromosome 2p15 and is located 69 kb upstream of *B3GNT2* and 158 kb downstream of *TMEM17*. In our study, we observed that subjects carrying GG of rs4672495 have lower proportion of severe RA activity (Table 4). The association between rs4672495 and AS was shown in several studies of people of European descent and a Korean populations [43, 44]. However, allele G of rs4672495 is associated with higher susceptibility of AS in the European population [43] but lower susceptibility of AS in the Korean population [44]. Therefore, further efforts are needed to dissect these associations related to rs4672495. Besides rs4672495, several SNPs on 2p15 have been associated with autoimmune diseases. A SNP on *B3GNT2* was reported to be a susceptibility marker for RA in a Japanese GWAS meta-analysis [21]. SNP rs6545946 could be a candidate polymorphism for Crohn's disease [45]. In addition, our previous study reported that an intergenic SNP on 2p15, rs10865331, was associated with susceptibility and disease severity of AS [29], and the data from GTEx indicated that it could influence the expression of the *B3GNT2* gene in whole blood. These evidences indicate the association of chromosomes 2p15 with certain autoimmune diseases, although the mechanism and functions leading to progression of RA and disease activity still need to be elucidated.

We also observed an association of rs11900673 in *B3GNT2* with the presence of ACPA, although it did not pass the threshold of significance for multiple testing. *B3GNT2* (UDP-GlcNAc:betaGal beta-1,3-N-acetylglucosaminyltransferase 2) is a major synthase for polylactosamine, which is the main polylactosamine synthase. *B3GNT2* loci were previously reported to be also associated with AS. In a 2010 animal study, Togayachi and coworkers investigated the function of *B3GNT2* in transgenic knockout mice [46]. They reported that β3GnT2 had strong activity *in vitro* toward oligosaccharide substrates with polylactosamine structures and tetraantennary *N*-glycans. A *B3gnt2* deficiency did not influence thymocyte development, and T or B cell development was not affected by reduced polylactosamine. Polylactosamine levels on two established immune co-stimulatory molecules, CD28 on T cell and CD19 on B cells, were lower in *B3gnt2*^−/−^ mice than in wild-type mice. These conditions made *B3gnt2*^−/−^ T cells more sensitive to induction of intracellular Ca^2+^ flux after stimulation with anti-CD3ε/CD28 antibodies and caused them to proliferate more strongly. B cells also showed hyperproliferation on BCR stimulation. This evidence indicates that *B3GNT2* has an important role in regulating lymphocyte activities.

According to a report by Okada et al., rs2841277 is located near several genes, including *PLD4* and *AHNAK2* [21]. Because the GTEx database includes limited cell types, rs2841277 did not show a significant association of eQTL with the *PLD4* gene in the current database. It was associated with *AHNAK2* expression in muscularis cells from the esophagus (Table 5). On searching HaploReg V4.1, rs2841277 was located 147 base-pairs (bp) upstream of *PLD4*, and it is involved in the changing chromatin status of primary T cells. Further functional studies are needed to validate the impact of rs2841277 on *PLD4* expression. On the other hand, rs4672495 showed a significantly associated eQTL with the *B3GNT2* gene in whole blood in the current database (Table 5).

There are some limitations to this study. Data of the controls from Taiwan Biobank was taken through Taiwan View website, which provides genotype information only. From Taiwan View, we obtained summary genotype counts, but not genotypes of individual subjects. Therefore, when investigating susceptibility of RA, we were able to compare genotype distributions between cases and controls merely. For the RA patients, some important confounding factors, such as disease duration and smoking, were unmeasured and their effects could not be adjusted for when we examined associations with disease activity and autoantibodies. Additionally, data of our patients was collected after taking medication for RA and the association between polymorphisms and disease activity might be influenced by medication. Furthermore, the power of statistical testing for association might be reduced by the modest sample size of RA patients collected in this study. Larger cohort studies and replication studies in Taiwanese populations are required to validate the findings in this report.

Accumulating studies showed effects of gene-gene interaction or combinational polymorphisms on susceptibility of complex diseases [47–49], including RA [50]. Currently, we examined association of individual SNPs with susceptibility of RA. Since we have only summary genotype counts of the controls, as mentioned in the discussion about limitation of this study, we were unable to investigate associations related to gene-gene interaction or combinational polymorphisms in this study. Furthermore, the investigation of associations with multiple polymorphisms generally needs large sample size. To investigate effect of gene-gene interaction on RA, we have to collect more samples with more informative genotypic and phenotypic data in our future work.

In summary, this study replicated an association of a SNP in *PLD4*, rs2841277, with susceptibility of RA and showed that an AS-associated SNP, rs4672495, is associated with disease activity in the Taiwanese population. Functional studies and examination of epigenetic factors that show how susceptibility genes actually affect RA should be conducted in the future.

## MATERIALS AND METHODS

### Study subjects

Patients were sequentially recruited from an RA clinic at Taichung Veterans General Hospital (TCVGH; Taichung, Taiwan). Our study conformed to the *Declaration of Helsinki*, and the design of the study and final report were performed with the approval of the institutional review board of TCVGH (No. CF15031A). We received informed consent from all subjects before any data were collected. Patients with RA who satisfied the American College of Rheumatology (ACR) revised criteria for RA in 1987 [51] or the ACR and European League Against Rheumatism (EULAR) classification criteria for RA in 2010 [52] were asked to participate in the study. In total, 334 RA patients were recruited from the Arthritis Clinic. RA was diagnosed by a qualified rheumatologist. Information of the 28-joint disease activity score (DAS28) was collected and used in the classification of disease activity. According to EULAR criteria, the disease activity was classified as remission, mild, moderate and severe, if DAS28 score was less than 2.6, between 2.6 and 3.2, between 3.2 and 5.1, or larger than 5.1, respectively [53].

The control group consists of 16,036 healthy subjects from the Taiwan Biobank (TWB) project [30], whose genotype counts can be accessed through the Taiwan View website (https://taiwanview.twbiobank.org.tw/index, data last accessed March 31, 2017).

### Candidate SNPs

As mentioned in INTRODUCTION, the SNPs investigated in this study were taken from Okada et al. (2012) [21] and Wen et al. (2014) [29]. Based on a meta-analysis of GWASs, Okada et al. (2012) reported nine SNPs associated with RA in a Japanese population [21]. Among these nine SNPs, rs2867461 (*ANXA3*) was excluded from the statistical analysis, because many subjects were unsuccessfully genotyped due to failure of polymerase chain reaction (PCR) amplification. On the other hand, Wen et al. (2014) examined seven SNPs from previous AS GWASs in a Taiwanese AS population [29]. Therefore, fifteen SNPs were analyzed in this study.

### DNA extraction

Peripheral venous blood was collected during medical surveillance, stored at 4°C, and processed the same day. Blood was centrifuged to separate serum and cells. DNA of blood cells was extracted by first treating them with 0.5% sodium dodecylsulfate-lysis buffer and then protease K (1 mg/ml) to digest nuclear proteins for 4 h at 60°C according to the manufacturer's procedures. Total DNA was harvested using a Gentra (Qiagen, Valencia, CA) extraction kit followed by 70% alcohol precipitation as recommended.

### Genotyping

Genotyping for the 15 SNPs was performed using the TaqMan Allelic Discrimination Assay (Applied Biosystems, Foster City, CA). PCR was performed in a 96-well microplate with an ABI9700 Thermal Cycler (Applied Biosystems, Foster City, CA). After the PCR, fluorescence was measured and analyzed using the System SDS software version 1.2.3 (Applied Biosystems, Foster City, CA).

The genotype counts of the 16,036 controls were obtained from Taiwan View (https://taiwanview.twbiobank.org.tw/index), which is a publicly accessible website developed by the TWB project. In the TWB project, two approaches were implemented to generate genotype data: SNP array (Affymetrix Axiom TWB genotype array) and whole genome sequencing (WGS). All the 16,036 controls have SNP array data and 997 of these controls also have WGS data. Ten of 15 SNPs analyzed in our study were contained in the SNP array and estimations of their genotypic and allelic distributions were based on the array data. The genotypic and allelic distributions of other 5 SNPs (rs2233434 (*NFKBIE*), rs3781913 (*PDE2A-ARAP1*), rs11209032, rs4672495, rs3734523 (*SLC17A2*)) were estimated by using the WGS data.

### SNP annotation data query

The GTEx Portal (http://www.gtexportal.org/home/), which includes a variety of tissue expression quantitative trait loci (eQTLs) was used to investigate associations between gene expression profiles and the SNPs.

### Statistical analysis

For each SNP, the Hardy-Weinberg equilibrium (HWE) in controls was evaluated by using a chi-squared (χ^2^) test.

A two-stage approach was used to investigate the associations of SNPs with RA susceptibility, RA activity, and autoantibodies. In the first stage, SNPs whose genotype/allele distributions are different between RA patients and controls, between severe and moderate patients, between ACPA (+) and ACPA (−) patients, or between RF (+) and RF (−) patients, were identified. All the comparisons in the first stage were based on Pearson's chi-squared test (or Fisher's exact test if needed) and the significance level of 0.05 was used. To deal with multiple testing, we calculate a q-value for each test, which is a false discovery rate (FDR)-based approach [54]. Any difference with q-value<0.1 was considered significant.

Significant association observed in the first stage was re-examined under different genetic models in the second stage. For RA susceptibility, associations under genotypic, dominant, and recessive models were evaluated by Pearson's chi-squared test and association under additive model was evaluated by Cochran-Armitage trend test. For other traits, the associations under various genetic models were assessed through multiple logistic regression models and were adjusted for age and gender. In analyses of the second stage, the significance level of 0.05 was used and the Bonferroni correction was implemented to deal with multiple testing.

All statistical analyses were implemented by using SAS 9.3 (SAS Institute, Cary, NC) for Windows.

## SUPPLEMENTARY MATERIALS TABLES




