# Supplementary material for: rs2841277 (PLD4) is associated with susceptibility and rs4672495 is associated with disease activity in rheumatoid arthritis
Source: Oncotarget. 2017 Jul 18;8(38):64180–90. doi: 10.18632/oncotarget.19419 (PMC5609993; doi:10.18632/oncotarget.19419)
Supplement: Supplementary file 1 [file oncotarget-08-64180-s001.pdf]

rs2841277 (*PLD4*) is associated with susceptibility and rs4672495 is associated with disease activity in rheumatoid arthritis

SUPPLEMENTARY MATERIALS

Supplementary Table 1: Comparisons of genotype and allele distributions between rheumatoid arthritis patients and controls

See Supplementary File 1

Supplementary Table 2: Comparisons of genotype and allele distributions between severe and moderate rheumatoid arthritis patients

See Supplementary File 1

Supplementary Table 3: Comparisons of genotype and allele distributions between ACPA (+) and ACPA (-) rheumatoid arthritis patients

See Supplementary File 1

Supplementary Table 4: Comparisons of genotype and allele distributions between RF (+) and RF (-) rheumatoid arthritis patients

See Supplementary File 1
