# Supplementary material for: rs2841277 (PLD4) is associated with susceptibility and rs4672495 is associated with disease activity in rheumatoid arthritis
Source: Oncotarget. 2017 Jul 18;8(38):64180–90. doi: 10.18632/oncotarget.19419 (PMC5609993; doi:10.18632/oncotarget.19419)
Supplement: Supplementary file 2 [file oncotarget-08-64180-s002.doc]

| **Supplementary Table 1: Comparisons of genotype and allele distributions between rheumatoid arthritis patients and controls.** | | | | | | | | | |
| --- | --- | --- | --- | --- | --- | --- | --- | --- | --- |
| **SNP** | **Genotype** | **Genotype frequencies** | | ***P* value**  **[q value]** |  | **Allele** | **Allele frequencies** | | ***P* value**  **[q value]** |
|  |  | **Case (%)** | **Control (%)** |  |  |  | **Case (%)** | **Control (%)** |  |
| *B3GNT2* | TT | 13 (3.9) | 484 (3.0) | 6.3E-01 |  | T | 121 (18.1) | 5684 (17.7) | 8.0E-01 |
| rs11900673 | CT | 95 (28.4) | 4716 (29.4) |  |  | C | 547 (81.9) | 26370 (82.3) |  |
|  | CC | 226 (67.7) | 10827 (67.6) |  |  |  |  |  |  |
| *CSF2* | AA | 23 (6.9) | 1051 (6.6) | 3.2E-01 |  | A | 184 (27.6) | 8138 (25.4) | 1.9E-01 |
| rs657075 | GA | 138 (41.4) | 6036 (37.6) |  |  | G | 482 (72.4) | 23914 (74.6) |  |
|  | GG | 172 (51.7) | 8939 (55.8) |  |  |  |  |  |  |
| *CD83* | CC | 14 (4.2) | 875 (5.5) | 3.4E-01 |  | C | 158 (23.7) | 7461 (23.3) | 8.2E-01 |
| rs12529514 | TC | 130 (38.9) | 5711 (35.6) |  |  | T | 510 (76.3) | 24581 (76.7) |  |
|  | TT | 190 (56.9) | 9435 (58.9) |  |  |  |  |  |  |
| *NFKBIE* | CC | 8 (2.4) | 22 (2.3) | 9.5E-01 |  | C | 105 (15.8) | 306 (16.1) | 8.3E-01 |
| rs2233434^†^ | TC | 89 (26.7) | 262 (27.6) |  |  | T | 561 (84.2) | 1592 (83.9) |  |
|  | TT | 236 (70.9) | 665 (70.1) |  |  |  |  |  |  |
| *ARID5B* | GG | 32 (9.6) | 1110 (6.9) | 1.3E-01 |  | G | 197 (29.5) | 8484 (26.5) | 8.1E-02 |
| rs10821944 | TG | 133 (39.8) | 6264 (39.1) |  |  | T | 471 (70.5) | 23552 (73.5) |  |
|  | TT | 169 (50.6) | 8644 (54.0) |  |  |  |  |  |  |
| *PDE2A-ARAP1* | CC | 26 (7.8) | 77 (7.7) | 7.7E-01 |  | C | 196 (29.3) | 561 (28.2) | 5.8E-01 |
| rs3781913^†^ | AC | 144 (43.1) | 407 (41.0) |  |  | A | 472 (70.7) | 1427 (71.8) |  |
|  | AA | 164 (49.1) | 510 (51.3) |  |  |  |  |  |  |
| *PLD4* | CC | 40 (12.0) | 2849 (17.8) | **8.3E-06** |  | C | 219 (32.9) | 13424 (41.9) | **2.9E-06** |
| rs2841277 | TC | 139 (41.7) | 7726 (48.2) | [0.0012] |  | T | 447 (67.1) | 18606 (58.1) | [0.0008] |
|  | TT | 154 (46.3) | 5440 (34.0) |  |  |  |  |  |  |
| *PTPN2* | GG | 41 (12.3) | 1472 (9.2) | 1.3E-01 |  | G | 222 (33.2) | 9619 (30.0) | 7.3E-02 |
| rs2847297 | AG | 140 (41.9) | 6675 (41.7) |  |  | A | 446 (66.8) | 22427 (70.0) |  |
|  | AA | 153 (45.8) | 7876 (49.1) |  |  |  |  |  |  |
|  | | | | | | | | | |

| **Supplementary Table 1: Comparisons of genotype and allele distributions between rheumatoid arthritis patients and controls. (continued)** | | | | | | | | | |
| --- | --- | --- | --- | --- | --- | --- | --- | --- | --- |
| **SNP** | **Genotype** | **Genotype frequencies** | | ***P* value**  **[q value]** |  | **Allele** | **Allele frequencies** | | ***P* value**  **[q value]** |
|  |  | **Case (%)** | **Control (%)** |  |  |  | **Case (%)** | **Control (%)** |  |
| rs11209032^†^ | AA | 72 (21.6) | 238 (23.9) | 1.2E-01 |  | A | 331 (49.7) | 970 (48.8) | 6.9E-01 |
|  | AG | 187 (56.2) | 494 (49.7) |  |  | G | 335 (50.3) | 1018 (51.2) |  |
|  | GG | 74 (22.2) | 262 (26.4) |  |  |  |  |  |  |
| rs4672495^†^ | GG | 6 (1.8) | 31 (3.1) | 4.1E-01 |  | G | 113 (16.9) | 347 (17.4) | 7.7E-01 |
|  | GT | 101 (30.2) | 285 (28.6) |  |  | T | 555 (83.1) | 1645 (82.6) |  |
|  | TT | 227 (68.0) | 680 (68.3) |  |  |  |  |  |  |
| rs10865331 | AA | 90 (27.0) | 3764 (23.5) | 2.3E-01 |  | A | 347 (51.9) | 15561 (48.6) | 8.6E-02 |
|  | AG | 167 (50.0) | 8033 (50.2) |  |  | G | 321 (48.1) | 16463 (51.4) |  |
|  | GG | 77 (23.0) | 4215 (26.3) |  |  |  |  |  |  |
| *ERAP1* | GG | 73 (21.9) | 3453 (21.6) | 1.9E-01 |  | G | 327 (49.0) | 14924 (46.6) | 2.3E-01 |
| rs27434 | GA | 181 (54.2) | 8018 (50.1) |  |  | A | 341 (51.0) | 17084 (53.4) |  |
|  | AA | 80 (23.9) | 4533 (28.3) |  |  |  |  |  |  |
| *SLC17A2* | AA | 1 (0.3) | 0 (0.0) | 2.5E-01 |  | A | 17 (2.6) | 54 (2.7) | 8.4E-01 |
| rs3734523^†^ | AG | 15 (4.5) | 54 (5.4) |  |  | G | 647 (97.4) | 1940 (97.3) |  |
|  | GG | 316 (95.2) | 943 (94.6) |  |  |  |  |  |  |
| rs13202464 | GG | 3 (0.9) | 87 (0.5) | 5.6E-01 |  | G | 54 (8.1) | 2290 (7.2) | 3.6E-01 |
|  | GA | 48 (14.4) | 2116 (13.2) |  |  | A | 614 (91.9) | 29720 (92.8) |  |
|  | AA | 283 (84.7) | 13802 (86.3) |  |  |  |  |  |  |
| rs13210693 | AA | 68 (20.4) | 3672 (23.0) | 5.3E-01 |  | A | 310 (46.4) | 15337 (47.9) | 4.4E-01 |
|  | AG | 174 (52.1) | 7993 (49.9) |  |  | G | 358 (53.6) | 16681 (52.1) |  |
|  | GG | 92 (27.5) | 4344 (27.1) |  |  |  |  |  |  |
| *P*-value < 0.05 was shown in bold. *Q*-value was shown when *p* < 0.05.  ^†^The distributions of genotypes and alleles in control group were estimated by using WGS data of 997 subjects. | | | | | | | | | |

| **Supplementary Table 2: Comparisons of genotype and allele distributions between severe and moderate rheumatoid arthritis patients.** | | | | | | | | | |
| --- | --- | --- | --- | --- | --- | --- | --- | --- | --- |
| **SNP** | **Genotype** | **Genotype frequencies** | | ***P* value**  **[q value]** |  | **Allele** | **Allele frequencies** | | ***P* value**  **[q value]** |
|  |  | **Severe (%)** | **Moderate (%)** |  |  |  | **Severe (%)** | **Moderate (%)** |  |
| *B3GNT2* | TT | 9 (3.4) | 1 (3.7) | 1.0E00 |  | T | 92 (17.5) | 9 (16.7) | 8.8E-01 |
| rs11900673 | CT | 74 (28.2) | 7 (25.9) |  |  | C | 434 (82.5) | 45 (83.3) |  |
|  | CC | 180 (68.4) | 19 (70.4) |  |  |  |  |  |  |
| *CSF2* | AA | 20 (7.6) | 1 (3.7) | 8.6E-01 |  | A | 142 (27.1) | 14 (25.9) | 8.5E-01 |
| rs657075 | GA | 102 (38.9) | 12 (44.4) |  |  | G | 382 (72.9) | 40 (74.1) |  |
|  | GG | 140 (53.5) | 14 (51.9) |  |  |  |  |  |  |
| *CD83* | CC | 11 (4.2) | 1 (3.7) | 6.3E-01 |  | C | 125 (23.8) | 10 (18.5) | 3.9E-01 |
| rs12529514 | TC | 103 (39.2) | 8 (29.6) |  |  | T | 401 (76.2) | 44 (81.5) |  |
|  | TT | 149 (56.6) | 18 (66.7) |  |  |  |  |  |  |
| *NFKBIE* | CC | 6 (2.3) | 1 (3.7) | 7.5E-01 |  | C | 81 (15.5) | 9 (16.7) | 8.2E-01 |
| rs2233434 | TC | 69 (26.3) | 7 (25.9) |  |  | T | 443 (84.5) | 45 (83.3) |  |
|  | TT | 187 (71.4) | 19 (70.4) |  |  |  |  |  |  |
| *ARID5B* | GG | 27 (10.3) | 1 (3.6) | 4.3E-01 |  | G | 152 (28.9) | 15 (27.8) | 8.6E-01 |
| rs10821944 | TG | 98 (37.2) | 13 (48.2) |  |  | T | 374 (71.1) | 39 (72.2) |  |
|  | TT | 138 (52.5) | 13 (48.2) |  |  |  |  |  |  |
| *PDE2A-ARAP1* | CC | 17 (6.5) | 3 (11.1) | 3.7E-01 |  | C | 150 (28.5) | 15 (27.8) | 9.1E-01 |
| rs3781913 | AC | 116 (44.1) | 9 (33.3) |  |  | A | 376 (71.5) | 39 (72.2) |  |
|  | AA | 130 (49.4) | 15 (55.6) |  |  |  |  |  |  |
| *PLD4* | CC | 32 (12.2) | 2 (7.4) | 2.2E-01 |  | C | 171 (32.6) | 20 (37.0) | 5.1E-01 |
| rs2841277 | TC | 107 (40.8) | 16 (59.3) |  |  | T | 353 (67.4) | 34 (63.0) |  |
|  | TT | 123 (47.0) | 9 (33.3) |  |  |  |  |  |  |
| *PTPN2* | GG | 31 (11.8) | 2 (7.4) | 6.5E-01 |  | G | 172 (32.7) | 18 (33.3) | 9.2E-01 |
| rs2847297 | AG | 110 (41.8) | 14 (51.9) |  |  | A | 354 (67.3) | 36 (66.7) |  |
|  | AA | 122 (46.4) | 11 (40.7) |  |  |  |  |  |  |
|  | | | | | | | | | |

| **Supplementary Table 2: Comparisons of genotype and allele distributions between severe and moderate rheumatoid arthritis patients. (continued)** | | | | | | | | | |
| --- | --- | --- | --- | --- | --- | --- | --- | --- | --- |
| **SNP** | **Genotype** | **Genotype frequencies** | | ***P* value**  **[q value]** |  | **Allele** | **Allele frequencies** | | ***P* value**  **[q value]** |
|  |  | **Severe (%)** | **Moderate (%)** |  |  |  | **Severe (%)** | **Moderate (%)** |  |
| rs11209032 | AA | 56 (21.4) | 11 (40.8) | **4.0E-02** |  | A | 259 (49.4) | 31 (57.4) | 2.6E-01 |
|  | AG | 147 (56.1) | 9 (33.3) | [0.54] |  | G | 265 (50.6) | 23 (42.6) |  |
|  | GG | 59 (22.5) | 7 (25.9) |  |  |  |  |  |  |
| rs4672495 | GG | 3 (1.1) | 3 (11.1) | **3.1E-03** |  | G | 87 (16.5) | 9 (16.7) | 9.8E-01 |
|  | GT | 81 (30.8) | 3 (11.1) | [0.084] |  | T | 439 (83.5) | 45 (83.3) |  |
|  | TT | 179 (68.1) | 21 (77.8) |  |  |  |  |  |  |
| rs10865331 | AA | 75 (28.5) | 8 (29.6) | 5.4E-01 |  | A | 282 (53.6) | 27 (50.0) | 6.1E-01 |
|  | AG | 132 (50.2) | 11 (40.8) |  |  | G | 244 (46.4) | 27 (50.0) |  |
|  | GG | 56 (21.3) | 8 (29.6) |  |  |  |  |  |  |
| *ERAP1* | GG | 54 (20.5) | 5 (18.5) | 8.3E-01 |  | G | 253 (48.1) | 24 (44.4) | 6.1E-01 |
| rs27434 | GA | 145 (55.1) | 14 (51.9) |  |  | A | 273 (51.9) | 30 (55.6) |  |
|  | AA | 64 (24.4) | 8 (29.6) |  |  |  |  |  |  |
| *SLC17A2* | AA | 0 (0.0) | 0 (0.0) | 6.2E-01 |  | A | 13 (2.5) | 0 (0.0) | 6.2E-01 |
| rs3734523 | AG | 13 (5.0) | 0 (0.0) |  |  | G | 509 (97.5) | 54 (100.0) |  |
|  | GG | 248 (95.0) | 27 (100.0) |  |  |  |  |  |  |
| rs13202464 | GG | 3 (1.1) | 0 (0.0) | 8.3E-01 |  | G | 44 (8.4) | 3 (5.6) | 6.1E-01 |
|  | GA | 38 (14.5) | 3 (11.1) |  |  | A | 482 (91.6) | 51 (94.4) |  |
|  | AA | 222 (84.4) | 24 (88.9) |  |  |  |  |  |  |
| rs13210693 | AA | 57 (21.7) | 5 (18.5) | 7.7E-01 |  | A | 241 (45.8) | 25 (46.3) | 9.5E-01 |
|  | AG | 127 (48.3) | 15 (56.6) |  |  | G | 285 (54.2) | 29 (53.7) |  |
|  | GG | 79 (30.0) | 7 (25.9) |  |  |  |  |  |  |
| *P*-value < 0.05 was shown in bold. *Q*-value was shown when *p* < 0.05. | | | | | | | | | |

| **Supplementary Table 3: Comparisons of genotype and allele distributions between ACPA (+) and ACPA (-) rheumatoid arthritis patients.** | | | | | | | | | |
| --- | --- | --- | --- | --- | --- | --- | --- | --- | --- |
| **SNP** | **Genotype** | **Genotype frequencies** | | ***P* value**  **[q value]** |  | **Allele** | **Allele frequencies** | | ***P* value**  **[q value]** |
|  |  | **ACPA (+) (%)** | **ACPA (-) (%)** |  |  |  | **ACPA (+) (%)** | **ACPA (-) (%)** |  |
| *B3GNT2* | TT | 10 (5.2) | 1 (1.4) | 1.2E-01 |  | T | 80 (20.7) | 18 (12.3) | **2.6E-02** |
| rs11900673 | CT | 60 (31.1) | 16 (21.9) |  |  | C | 306 (79.3) | 128 (87.7) | [0.50] |
|  | CC | 123 (63.7) | 56 (76.7) |  |  |  |  |  |  |
| *CSF2* | AA | 11 (5.7) | 5 (6.8) | 7.7E-01 |  | A | 102 (26.6) | 37 (25.3) | 7.8E-01 |
| rs657075 | GA | 80 (41.7) | 27 (37.0) |  |  | G | 282 (73.4) | 109 (74.7) |  |
|  | GG | 101 (52.6) | 41 (56.2) |  |  |  |  |  |  |
| *CD83* | CC | 9 (4.6) | 2 (2.7) | 8.6E-01 |  | C | 88 (22.8) | 30 (20.5) | 5.8E-01 |
| rs12529514 | TC | 70 (36.3) | 26 (35.6) |  |  | T | 298 (77.2) | 116 (79.5) |  |
|  | TT | 114 (59.1) | 45 (61.7) |  |  |  |  |  |  |
| *NFKBIE* | CC | 6 (3.1) | 1 (1.4) | 7.5E-01 |  | C | 68 (17.6) | 21 (14.6) | 4.1E-01 |
| rs2233434 | TC | 56 (29.0) | 19 (26.4) |  |  | T | 318 (82.4) | 123 (85.4) |  |
|  | TT | 131 (67.9) | 52 (72.2) |  |  |  |  |  |  |
| *ARID5B* | GG | 16 (8.3) | 10 (13.7) | 3.8E-01 |  | G | 111 (28.8) | 50 (34.2) | 2.2E-01 |
| rs10821944 | TG | 79 (40.9) | 30 (41.1) |  |  | T | 275 (71.2) | 96 (65.8) |  |
|  | TT | 98 (50.8) | 33 (45.2) |  |  |  |  |  |  |
| *PDE2A-ARAP1* | CC | 15 (7.8) | 5 (6.9) | 6.1E-01 |  | C | 112 (29.0) | 46 (31.5) | 5.7E-01 |
| rs3781913 | AC | 82 (42.5) | 36 (49.3) |  |  | A | 274 (71.0) | 100 (68.5) |  |
|  | AA | 96 (49.7) | 32 (43.8) |  |  |  |  |  |  |
| *PLD4* | CC | 17 (8.8) | 12 (16.7) | 9.6E-02 |  | C | 118 (30.6) | 58 (40.3) | **3.5E-02** |
| rs2841277 | TC | 84 (43.5) | 34 (47.2) |  |  | T | 268 (69.4) | 86 (59.7) | [0.50] |
|  | TT | 92 (47.7) | 26 (36.1) |  |  |  |  |  |  |
| *PTPN2* | GG | 19 (9.8) | 10 (13.7) | 1.8E-01 |  | G | 125 (32.4) | 44 (30.1) | 6.2E-01 |
| rs2847297 | AG | 87 (45.1) | 24 (32.9) |  |  | A | 261 (67.6) | 102 (69.9) |  |
|  | AA | 87 (45.1) | 39 (53.4) |  |  |  |  |  |  |
|  | | | | | | | | | |

| **Supplementary Table 3: Comparisons of genotype and allele distributions between ACPA (+) and ACPA (-) rheumatoid arthritis patients. (continued)** | | | | | | | | | |
| --- | --- | --- | --- | --- | --- | --- | --- | --- | --- |
| **SNP** | **Genotype** | **Genotype frequencies** | | ***P* value**  **[q value]** |  | **Allele** | **Allele frequencies** | | ***P* value**  **[q value]** |
|  |  | **ACPA (+) (%)** | **ACPA (-) (%)** |  |  |  | **ACPA (+) (%)** | **ACPA (-) (%)** |  |
| rs11209032 | AA | 42 (21.9) | 19 (26.0) | 7.7E-01 |  | A | 194 (50.5) | 78 (53.4) | 5.5E-01 |
|  | AG | 110 (57.3) | 40 (54.8) |  |  | G | 190 (49.5) | 68 (46.6) |  |
|  | GG | 40 (20.8) | 14 (19.2) |  |  |  |  |  |  |
| rs4672495 | GG | 4 (2.1) | 1 (1.4) | 9.6E-01 |  | G | 69 (17.9) | 26 (17.8) | 9.9E-01 |
|  | GT | 61 (31.6) | 24 (32.9) |  |  | T | 317 (82.1) | 120 (82.2) |  |
|  | TT | 128 (66.3) | 48 (65.7) |  |  |  |  |  |  |
| rs10865331 | AA | 52 (26.9) | 22 (30.1) | 8.5E-01 |  | A | 201 (52.1) | 80 (54.8) | 5.7E-01 |
|  | AG | 97 (50.3) | 36 (49.3) |  |  | G | 185 (47.9) | 66 (45.2) |  |
|  | GG | 44 (22.8) | 15 (20.6) |  |  |  |  |  |  |
| *ERAP1* | GG | 44 (22.8) | 12 (16.4) | 5.2E-01 |  | G | 189 (49.0) | 65 (44.5) | 3.6E-01 |
| rs27434 | GA | 101 (52.3) | 41 (56.2) |  |  | A | 197 (51.0) | 81 (55.5) |  |
|  | AA | 48 (24.9) | 20 (27.4) |  |  |  |  |  |  |
| *SLC17A2* | AA | 0 (0.0) | 0 (0.0) | 2.9E-01 |  | A | 9 (2.3) | 1 (0.7) | 3.0E-01 |
| rs3734523 | AG | 9 (4.7) | 1 (1.4) |  |  | G | 375 (97.7) | 145 (99.3) |  |
|  | GG | 183 (95.3) | 72 (98.6) |  |  |  |  |  |  |
| rs13202464 | GG | 2 (1.0) | 1 (1.4) | 1.1E-01 |  | G | 26 (6.7) | 17 (11.6) | 6.4E-02 |
|  | GA | 22 (11.4) | 15 (20.5) |  |  | A | 360 (93.3) | 129 (88.4) |  |
|  | AA | 169 (87.6) | 57 (78.1) |  |  |  |  |  |  |
| rs13210693 | AA | 40 (20.7) | 19 (26.0) | 5.5E-01 |  | A | 178 (46.1) | 75 (51.4) | 2.8E-01 |
|  | AG | 98 (50.8) | 37 (50.7) |  |  | G | 208 (53.9) | 71 (48.6) |  |
|  | GG | 55 (28.5) | 17 (23.3) |  |  |  |  |  |  |
| *P*-value < 0.05 was shown in bold. *Q*-value was shown when *p* < 0.05. | | | | | | | | | |

| **Supplementary Table 4: Comparisons of genotype and allele distributions between RF (+) and RF (-) rheumatoid arthritis patients.** | | | | | | | | | |
| --- | --- | --- | --- | --- | --- | --- | --- | --- | --- |
| **SNP** | **Genotype** | **Genotype frequencies** | | ***P* value**  **[q value]** |  | **Allele** | **Allele frequencies** | | ***P* value**  **[q value]** |
|  |  | **RF (+) (%)** | **RF (-) (%)** |  |  |  | **RF (+) (%)** | **RF (-) (%)** |  |
| *B3GNT2* | TT | 10 (3.8) | 3 (4.3) | 1.2E-01 |  | T | 101 (19.2) | 19 (13.6) | 1.2E-01 |
| rs11900673 | CT | 81 (30.8) | 13 (18.6) |  |  | C | 425 (80.8) | 121 (86.4) |  |
|  | CC | 172 (65.4) | 54 (77.1) |  |  |  |  |  |  |
| *CSF2* | AA | 18 (6.9) | 5 (7.1) | 2.4E-01 |  | A | 151 (28.8) | 33 (23.6) | 2.2E-01 |
| rs657075 | GA | 115 (43.9) | 23 (32.9) |  |  | G | 373 (71.2) | 107 (76.4) |  |
|  | GG | 129 (49.2) | 42 (60.0) |  |  |  |  |  |  |
| *CD83* | CC | 13 (5.0) | 1 (1.4) | 3.4E-01 |  | C | 131 (24.9) | 27 (19.3) | 1.6E-01 |
| rs12529514 | TC | 105 (39.9) | 25 (35.7) |  |  | T | 395 (75.1) | 113 (80.7) |  |
|  | TT | 145 (55.1) | 44 (62.9) |  |  |  |  |  |  |
| *NFKBIE* | CC | 8 (3.1) | 0 (0.0) | 4.7E-01 |  | C | 85 (16.2) | 19 (13.8) | 4.9E-01 |
| rs2233434 | TC | 69 (26.2) | 19 (27.5) |  |  | T | 441 (83.8) | 119 (86.2) |  |
|  | TT | 186 (70.7) | 50 (72.5) |  |  |  |  |  |  |
| *ARID5B* | GG | 23 (8.8) | 9 (12.9) | 1.9E-01 |  | G | 146 (27.8) | 50 (35.7) | 6.6E-02 |
| rs10821944 | TG | 100 (38.0) | 32 (45.7) |  |  | T | 380 (72.2) | 90 (64.3) |  |
|  | TT | 140 (53.2) | 29 (41.4) |  |  |  |  |  |  |
| *PDE2A-ARAP1* | CC | 21 (8.0) | 5 (7.2) | 2.9E-01 |  | C | 150 (28.5) | 46 (32.9) | 3.2E-01 |
| rs3781913 | AC | 108 (41.1) | 36 (51.4) |  |  | A | 376 (71.5) | 94 (67.1) |  |
|  | AA | 134 (50.9) | 29 (41.4) |  |  |  |  |  |  |
| *PLD4* | CC | 30 (11.5) | 10 (14.2) | 7.3E-01 |  | C | 168 (32.1) | 50 (35.7) | 4.1E-01 |
| rs2841277 | TC | 108 (41.2) | 30 (42.9) |  |  | T | 356 (67.9) | 90 (64.3) |  |
|  | TT | 124 (47.3) | 30 (42.9) |  |  |  |  |  |  |
| *PTPN2* | GG | 30 (11.4) | 11 (15.7) | 5.0E-01 |  | G | 174 (33.1) | 48 (34.3) | 7.9E-01 |
| rs2847297 | AG | 114 (43.4) | 26 (37.1) |  |  | A | 352 (66.9) | 92 (65.7) |  |
|  | AA | 119 (45.2) | 33 (47.2) |  |  |  |  |  |  |
|  | | | | | | | | | |

| **Supplementary Table 4: Comparisons of genotype and allele distributions between RF (+) and RF (-) rheumatoid arthritis patients. (continued)** | | | | | | | | | |
| --- | --- | --- | --- | --- | --- | --- | --- | --- | --- |
| **SNP** | **Genotype** | **Genotype frequencies** | | ***P* value**  **[q value]** |  | **Allele** | **Allele frequencies** | | ***P* value**  **[q value]** |
|  |  | **RF (+) (%)** | **RF (-) (%)** |  |  |  | **RF (+) (%)** | **RF (-) (%)** |  |
| rs11209032 | AA | 56 (21.4) | 16 (22.9) | 9.6E-01 |  | A | 259 (49.4) | 71 (50.7) | 7.9E-01 |
|  | AG | 147 (56.1) | 39 (55.7) |  |  | G | 265 (50.6) | 69 (49.3) |  |
|  | GG | 59 (22.5) | 15 (21.4) |  |  |  |  |  |  |
| rs4672495 | GG | 5 (1.9) | 1 (1.4) | 1.0E00 |  | G | 90 (17.1) | 23 (16.4) | 8.5E-01 |
|  | GT | 80 (30.4) | 21 (30.0) |  |  | T | 436 (82.9) | 117 (83.6) |  |
|  | TT | 178 (67.7) | 48 (68.6) |  |  |  |  |  |  |
| rs10865331 | AA | 68 (25.9) | 21 (30.0) | 5.4E-01 |  | A | 272 (51.7) | 73 (52.1) | 9.3E-01 |
|  | AG | 136 (51.7) | 31 (44.3) |  |  | G | 254 (48.3) | 67 (47.9) |  |
|  | GG | 59 (22.4) | 18 (25.7) |  |  |  |  |  |  |
| *ERAP1* | GG | 61 (23.2) | 11 (15.7) | 3.3E-01 |  | G | 264 (50.2) | 61 (43.6) | 1.6E-01 |
| rs27434 | GA | 142 (54.0) | 39 (55.7) |  |  | A | 262 (49.8) | 79 (56.4) |  |
|  | AA | 60 (22.8) | 20 (28.6) |  |  |  |  |  |  |
| *SLC17A2* | AA | 1 (0.4) | 0 (0.0) | 6.3E-01 |  | A | 13 (2.5) | 4 (2.9) | 7.7E-01 |
| rs3734523 | AG | 11 (4.2) | 4 (5.7) |  |  | G | 509 (97.5) | 136 (97.1) |  |
|  | GG | 249 (95.4) | 66 (94.3) |  |  |  |  |  |  |
| rs13202464 | GG | 3 (1.1) | 0 (0.0) | 1.6E-01 |  | G | 39 (7.4) | 15 (10.7) | 2.0E-01 |
|  | GA | 33 (12.6) | 15 (21.4) |  |  | A | 487 (92.6) | 125 (89.3) |  |
|  | AA | 227 (86.3) | 55 (78.6) |  |  |  |  |  |  |
| rs13210693 | AA | 49 (18.6) | 19 (27.2) | 2.0E-01 |  | A | 235 (44.7) | 74 (52.9) | 8.5E-02 |
|  | AG | 137 (52.1) | 36 (51.4) |  |  | G | 291 (55.3) | 66 (47.1) |  |
|  | GG | 77 (29.3) | 15 (21.4) |  |  |  |  |  |  |
| *P*-value < 0.05 was shown in bold. *Q*-value was shown when *p* < 0.05. | | | | | | | | | |
